# A Bioactive Compound from *Sanguisorba officinalis* L. Inhibits Cell Proliferation and Induces Cell Death in 5-Fluorouracil-Sensitive/Resistant Colorectal Cancer Cells

**DOI:** 10.3390/molecules26133843

**Published:** 2021-06-24

**Authors:** Weijia Zhang, Chang Peng, Xue Shen, Yuemei Yuan, Wei Zhang, Chunjuan Yang, Meicun Yao

**Affiliations:** 1 School of Pharmaceutical Sciences, Sun Yat-sen University, Guangzhou 510006, China; zhangwj66@mail2.sysu.edu.cn (W.Z.); shenx27@mail2.sysu.edu.cn (X.S.); 2 School of Pharmaceutical Sciences (Shenzhen), Sun Yat-sen University, Shenzhen 518107, China; pengch56@mail2.sysu.edu.cn; 3 School of Ecology, Sun Yat-sen University, 6# Ming De Yuan, Guangzhou University City, Guangzhou 510006, China; yuanym@mail.sysu.edu.cn; 4 State Key Laboratory of Quality Research in Chinese Medicines, Macau University of Science and Technology, Taipa, Macau, China; wzhang@must.edu.mo; 5 Department of Pharmaceutical Analysis and Analytical Chemistry, College of Pharmacy Harbin Medical University, Harbin 150081, China

**Keywords:** colorectal cancer, AGE, apoptosis, autophagy, Wnt/β-catenin signaling pathway

## Abstract

Colorectal cancer (CRC) is one of the most common cancer in the world. The first line chemotherapeutic agent, 5-fluorouracil (5-FU), plays a predominant role in the clinical treatment of CRC. However, with the wide use of 5-FU, more and more CRC patients have been obtaining drug resistance to 5-FU, which leads to a large amount of treatment failures. One of the effective strategies to overcome this obstacle is to find bioactive natural products from traditional medicine. In our previous work, *Sanguisorba officinalis* L. was found to exert a strong anti-proliferative activity against 5-FU-senstive/resistant CRC cells. Therefore, several compounds were isolated from this herb and screened for their anti-CRC effects to find promising compounds. Among them, a triterpenoid compound named 3β-[(α-l-arabinopyranosyl) oxy]-urs-12,18(19)-dien-28-oic acid β-d-glucopyranosyl ester (AGE), showed strong activity against both 5-FU-senstive and resistant CRC cells. In order to further study the mechanism of AGE on CRC cells, flow cytometer analysis, mitochondrial membrane potential (MMP) measurement, Western blotting, and RT-PCR assays were performed. Results demonstrated that AGE induced cell death by apoptosis pathway and autophagy, and inhibited cell proliferation via cell cycle arrest in G0-G1 phase mediated by Wnt signaling pathway. Therefore, AGE may be a potential bioactive compound for CRC treatment in clinic.

## 1. Introduction

Colorectal cancer (CRC) is one of the most commonly diagnosed cancers in the world. According to the cancer statistics in 2019, CRC is the second most common cancer in males and the third in females [1]. Surgery and chemotherapy are two main prescriptions for CRC treatment. Until now, 5-fluorouracil (5-FU) remains the mainstay for chemotherapy of CRC [2,3]. However, more and more patients have developed drug resistance due to 5-FU-based chemotherapies [4], leading to failure in CRC treatment. Therefore, it is of prominent importance to develop novel agents to treat CRC effectively. An attractive strategy is to find bioactive compounds from natural plants or Chinese medicine.

*Sanguisorba officinalis* L., as a traditional Chinese medicine, has a variety of biological activities, such as anti-inflammatory, anti-oxidative, and anti-cancer activities [5,6,7]. Our previous study had reported that *Sanguisorba officinalis* L. aqueous extract significantly induced CRC cells’ apoptosis and autophagy, and synergistically enhanced 5-FU cytotoxicity in CRC cells (HCT116 and RKO cells) [8]. Fortunately, our previous data showed that *Sanguisorba officinalis* L. also strongly suppressed the 5-FU-resistant CRC cells. Therefore, a series of compounds were isolated from *Sanguisorba officinalis* L. by Yang’s team [9] and were measured for anti-proliferation effects on 5-FU-sensitive/resistant CRC cells. A bioactive triterpenoid compound from *Sanguisorba officinalis* L., named 3β-[(α-l-arabinopyranosyl) oxy]-urs-12,18(19)-dien-28-oic acid β-d-glucopyranosyl ester (AGE) was found to suppress both 5-FU-sensitive (RKO-P and HCT15-P) and resistant (RKO-R and HCT15-R) CRC cells proliferation. However, the potential function mechanisms have not been elucidated to date.

Based on continuous research, the apoptosis pathway, which belongs to the programmed cell death Ⅰ process, plays an essential role in cancer therapy. Additionally, accumulating evidence intimates that, not only cell apoptosis, but also autophagy, an autophagic cell death belonging to the programmed cell death Ⅱ process, is involved in anti-cancer [10,11]. The enforced over-activation of autophagy can lead to cell death, which has been confirmed by substantial studies [12]. In addition, the canonical Wnt/β-catenin signaling pathway is one of the signaling pathways highly related to cancer. Reports suggest that excessive activation of the Wnt/β-catenin signaling pathway causes progression in cancer development and chemo-resistance in various cancers, especially for CRC [13,14,15]. The abnormal activation of the Wnt pathway can lead to increasing downstream gene expression, such as *MYC* and *CCND1* genes, which will affect cell proliferation and cell cycle, etc. Consequently, targeting the apoptosis pathway, autophagy, and Wnt signaling pathway may allow us to uncover the function mechanism of AGE.

In this study, we aimed to study the effects of the AGE on 5-FU sensitive/resistant CRC cell proliferations and cell deaths, then elucidated the potential mechanisms. The AGE compound may be a potential agent to treat 5-FU-sensitive/resistant CRC patients in clinics, and improve the clinic outcome.

## 2. Results

### 2.1. AGE Inhibited Cells Proliferation and Caused Cells Death

**Figure 1:**
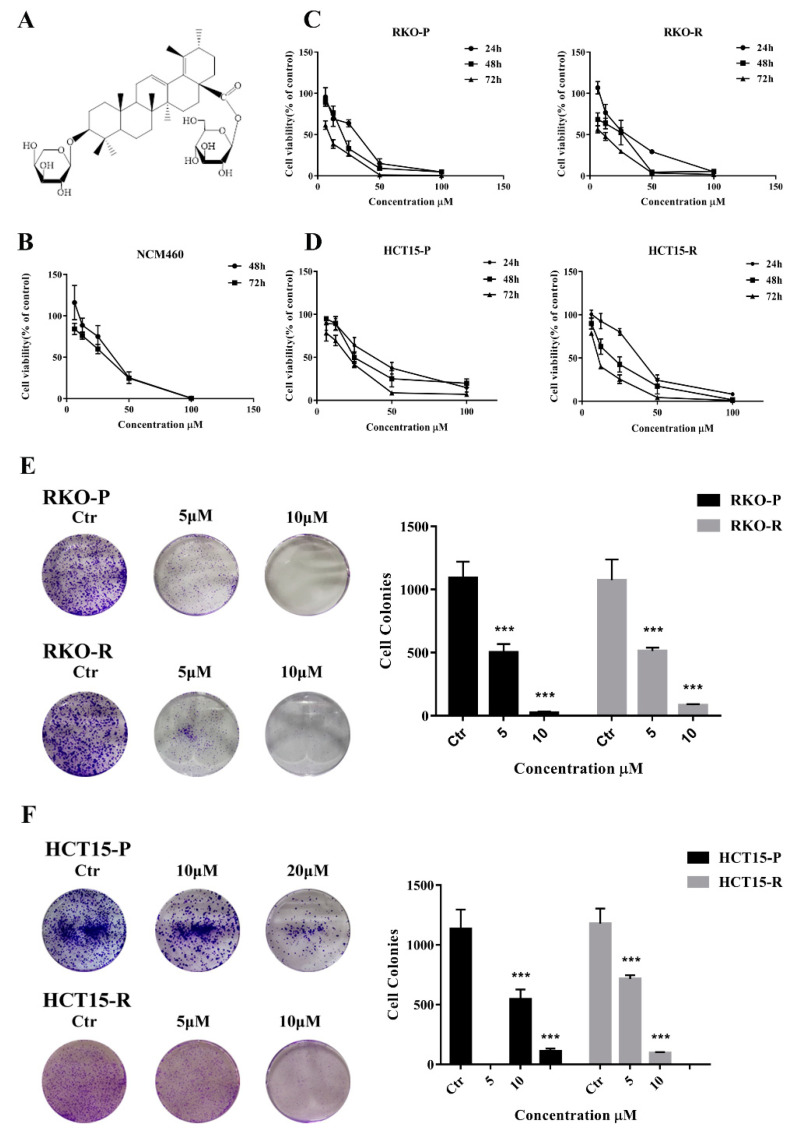
The effect of AGE on the proliferative activities against 5-FU-sensitive/resistant CRC cell lines. (**A**): The structure of AGE compound. (**B**): NCM460 cell was treated AGE after 48 h and 72 h, then cytotoxicity was determined by CCK8. (**C**,**D**): Cells were treated with AGE after 24 h, 48 h, and 72 h, then cell viability was measured with a CCK8 assay. (**E**,**F**): After nine days of treatment, a cell colony assay was performed to detect the effect of AGE on cell proliferation for the long term. Results are expressed as mean ± SD (*n* = 3). *** *p* < 0.001, compared with Control (Ctr) group.

In our previous study, the *Sanguisorba officinalis* L. had strong anti-CRC activity, including 5-FU resistant cells (Figure S1). Therefore, in this study, a series of products isolated from *Sanguisorba officinalis* L. were determined for the cytotoxic effects on the CRC cells by a CCK8 assay (Figure S2). The results showed that the AGE (Figure 1A) predominantly suppressed the cell proliferation in dose-dependence (Figure 1C,D). The IC_50_ values of AGE at 72 h in RKO-P and HCT15-P cells were 9.15 and 17.10 μM, respectively. Interestingly, the activity of AGE against 5-FU resistant cells was stronger than 5-FU sensitive cells. The IC_50_ values of RKO-R and HCT15-R cells were 6.49 and 10.99 μM, respectively (Table 1), while the IC_50_ for NCM460 cell (a kind of human normal intestinal epithelial cell) was 28.18 μM, much higher than all of CRC cell lines (Figure 1B and Table 1). There were IC50 values of 5-FU for CRC cell lines (Figure S3). The IC50 value of 5-FU resistant cells is over 20 times larger than that of sensitive cells, which indicated that the cell model was successfully established. Moreover, colony a formation assay was conducted to assess the effect of AGE on cells proliferation in the long term. The cell colony growth was repressed by AGE in a dose-dependent manner (Figure 1E,F). Taken together, these results showed that AGE implicitly restrained the CRC cell lines, including 5-FU resistant cells.

### 2.2. AGE Initiated Cells Intracellular Apoptotic Pathway

**Figure 2:**
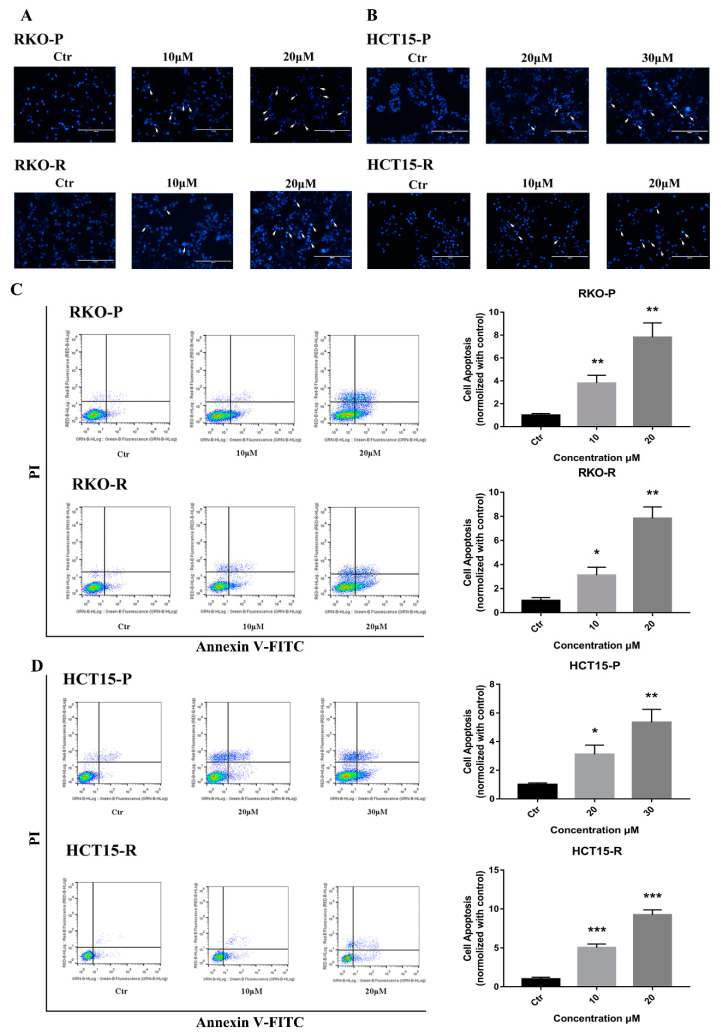
AGE induced apoptosis in 5-FU-sensitive/resistant CRC cell lines. (**A**,**B**): Cells were incubated with Hochst 33258 after 48 h of AGE treatment, then observed under a fluorescence microscope (200×). Arrow refers to condensed and bright chromatins. (**C**,**D**): Cells were treated with Annexin V-FITC/PI after 48 h of AGE incubation and detected by flow cytometry. The results of cell apoptosis normalized with control group are on the right bars. Results are expressed as mean ± SD (*n* = 3). * *p* < 0.05, ** *p* < 0.01, *** *p* < 0.001, compared with Control (Ctr) group.

**Figure 3:**
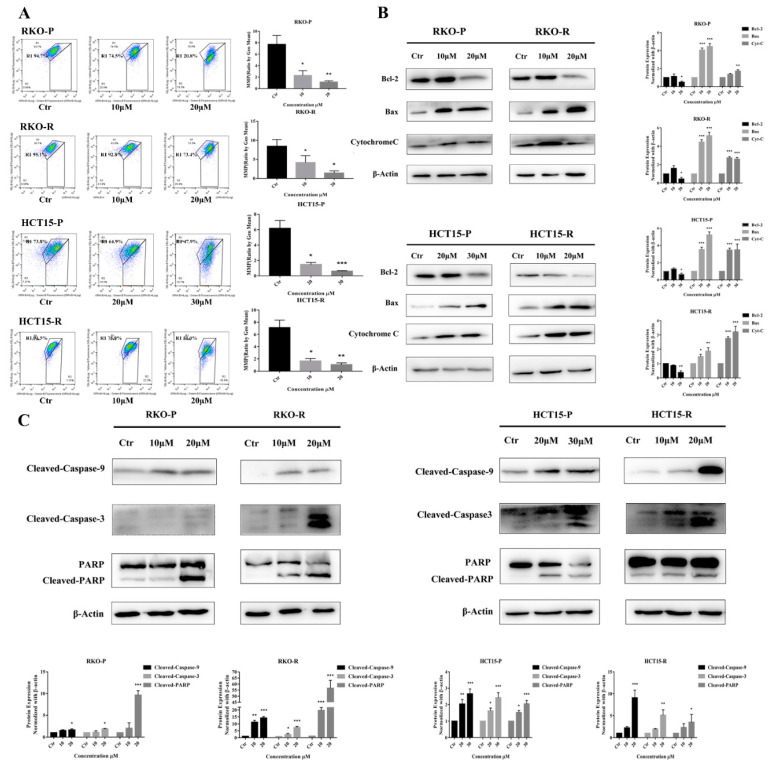
AGE induced apoptosis via mitochondrial-caspase-dependent pathway in 5-FU-sensitive/resistant CRC cell lines. (**A**): The MMP levels of cell lines were determined by JC-1 after 24 h of AGE treatment and measured by mean light intensity of red-green fluorescence. The results of MMP levels are on the right. (**B**): Following the treatment with AGE for 24 h, the expression levels of Bcl-2, Bax, and Cytochrome C proteins were determined by Western Blot and analyzed by β-Actin. The results are presented in right histograms. (**C**): Effects of 24 h treatment with AGE on the expression levels of cleaved-caspase-9, cleaved-caspase-3 and cleaved-PARP proteins in 5-FU-sensitive/resistant cell lines. The analysis results are shown below. Results are expressed as mean ± SD (*n* = 3). * *p* < 0.05, ** *p* < 0.01, *** *p* < 0.001, compared with Ctr group.

According to the CCK8 determination results, 10 μM and 20 μM AGE dosages were used for RKO-P/RKO-R and HCT15-R cell lines, and 20 μM and 30 μM were for HCT15-P cell line in all of the following experiments for 24 h or 48 h treatment. Under these conditions, AGE is able to keep most cells alive, and show obvious pharmacological effects. The Hoechst 33258 assay results showed that condensed and bright chromatins were observed in cells, elucidating that apoptosis was evoked by AGE in 48 h (Figure 2A,B). To investigate the apoptosis effects, an Annexin V-FITC/PI assay was conducted. The consequences demonstrated that the cell apoptotic rate was increased in the wake of AGE dosages after 48 h (Figure 2C,D). The percentages of apoptotic cells by high-dose were 22.95%, 20.48%, 15.28%, and 31.39% for RKO-P/RKO-R and HCT15-P/HCT15-R cells, respectively. Next, a JC-1 kit was used to ascertain whether the cells’ mitochondrial membrane permeability was enhanced. The results suggested that AGE caused all of the cells’ MMP to increase, representing that mitochondrial depolarization might be triggered after 24 h treatment (Figure 3A). The RKO-P cell ΔΨm declined from 7.71 to 1.139, and the RKO-R cell ΔΨm from 8.455 to 1.43. The HCT15-P cell ΔΨm declined from 6.178 to 0.61, and the HTC15-R cell ΔΨm from 7.132 to 1.09.

According to the above flow cytometry results, we supposed that cell apoptosis was prompted by the intracellular mitochondrial apoptosis pathway. To confirm this hypothesis, proteins related to the apoptosis pathway were assayed. We found that the expression of anti-apoptosis Bcl-2 protein decreased at a high drug concentration, but increased or remained constant at a low dose. In contrast, the expression of pro-apoptotic Bax protein was strengthened (Figure 3B). The alteration of these two proteins expression and cells’ MMP changes could result in enhancing the mitochondrial membrane permeability, and increasing Cytochrome C (Cyt C) release from the mitochondrion. Then, protein expression levels of Cyt C were measured in all of these cell lines. After 24 h AGE treatment, the Cyt C protein expression levels were apparently increased (Figure 3B). Based on the fact that the above proteins expression had changed, the caspase-dependent apoptosis pathway may be activated. Data also demonstrated that cleaved-caspase-9 was up-regulated by AGE treatment, which caused self-cleavage of its downstream executioner caspase-3. The cleaved-caspase-3 expression was increased in a dose-dependent manner, which further led to cleaved-PARP protein raising (Figure 3C). In conclusion, the compound AGE could induce CRC 5-FU sensitive/resistant cells apoptosis through mitochondria-caspase-dependent pathway.

### 2.3. AGE Elicited Autophagy

**Figure 4:**
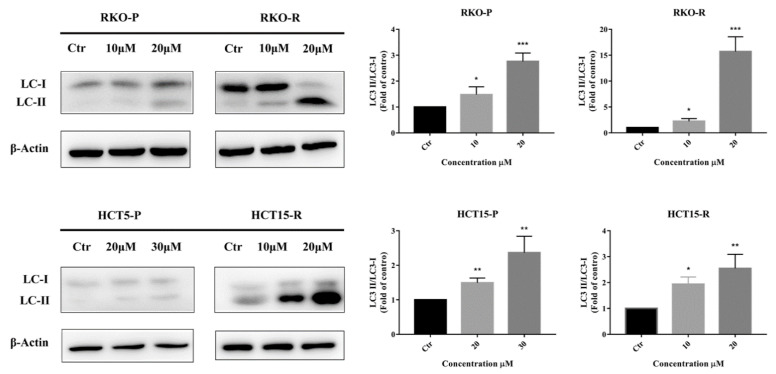
AGE activated autophagy pathway in 5-FU-sensitive/resistant CRC cell lines. Expression levels of LC3 Ⅱ and LC3 Ⅰ proteins were analyzed and, respectively, normalized by β-Actin after 24 h of AGE treatment. The ratio of LC3 Ⅱ to LC3 Ⅰ is shown in the right histogram. Results are expressed as mean ± SD (*n* = 3). * *p* < 0.05, ** *p* < 0.01, *** *p* < 0.001, compared with Ctr group.

In addition to cell apoptosis, cell autophagy is also a cause of cell death. Therefore, the expressions of protein and genes related to autophagy were also measured in this study. The most representative protein in cell autophagy program is LC 3 protein, made up of LC 3Ⅰ and LC 3Ⅱ [12]. The ratio of LC 3Ⅱ/LC 3Ⅰ protein is related to the degree of autophagy. After AGE treatment, intracellular LC 3Ⅱ/LC 3Ⅰ protein ratio was significantly increased, indicating that cell autophagy was activated (Figure 4). Therefore, AGE might activate cell autophagy, which could lead to cell death. 

### 2.4. AGE Arrested Cell Cycle at G0-G1 Phase

**Figure 5:**
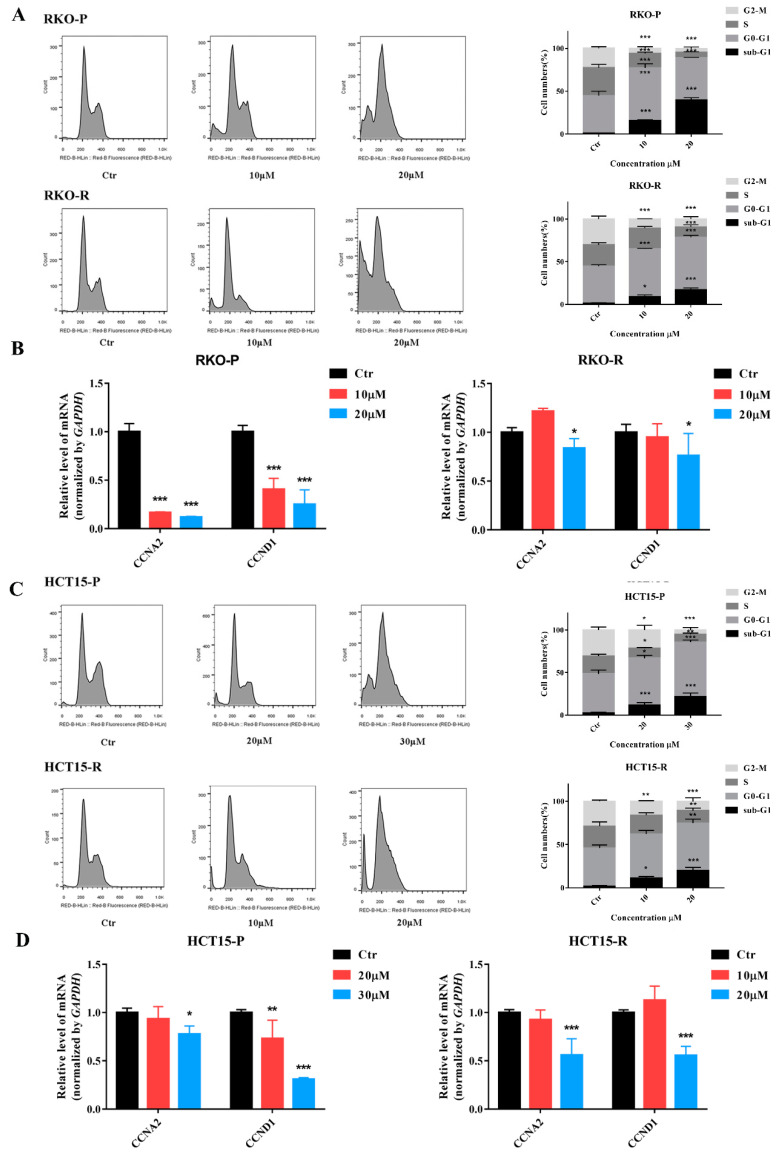
AGE arrested cell cycle at the G0-G1 phase in 5-FU-sensitive/resistant CRC cell lines. (**A**,**C**): The cell cycle was determined by flow cytometry after cells stained with PI/RNase. The results of the cell cycle are shown on the right. (**B**,**D**): Two cell cycle genes, *CCNA2* and *CCND1*, are measured by RT-PCR. The data was analyzed using a 2^−ΔΔCt^ approach. Results are expressed as mean ± SD (*n* = 3). * *p* < 0.05, ** *p* < 0.01, *** *p* < 0.001, compared with Ctr group.

To verify whether cell cycle arrest was involved in the cell apoptosis and anti-proliferation effects of AGE, all cell lines were treated with AGE for 48 h. Data represented that the sub-G1 phase was raised, which meant cells were undergoing apoptosis or necrosis. Additionally, most cells were arrested in the G0-G1 phase after AGE treatment (Figure 5A,C). The proportion of RKO-P and RKO-R cells at the G0-G1 phase was altered from 40.1% and 42.26% to 65.0 and 63.2%, respectively. However, for groups of high AGE doses in RKO-P cells, the G0-G1 phase possessed 49.9%, which caused increased cells in sub-G1 phase and decreased ratios of other phases. Additionally, the proportion of HCT15-P and HCT15-R cells at G0-G1 phase was changed from 46.0% and 44.1% to 64.1% and 54.7%, respectively.

To further authenticate that the cell cycle was arrested in the G0-G1 phase, RT-PCR was performed to determine expression levels of *CCNA2* (Cyclin A2) and *CCND1* (Cyclin D1). Both genes promote transition through G1/S and G2/M [16,17]. The results showed that *CCNA2* and *CCND1* gene expression levels were clearly reduced, dose dependently (Figure 5B,D).

### 2.5. AGE Blocked Wnt/β-Catenin Signaling Pathway

**Figure 6:**
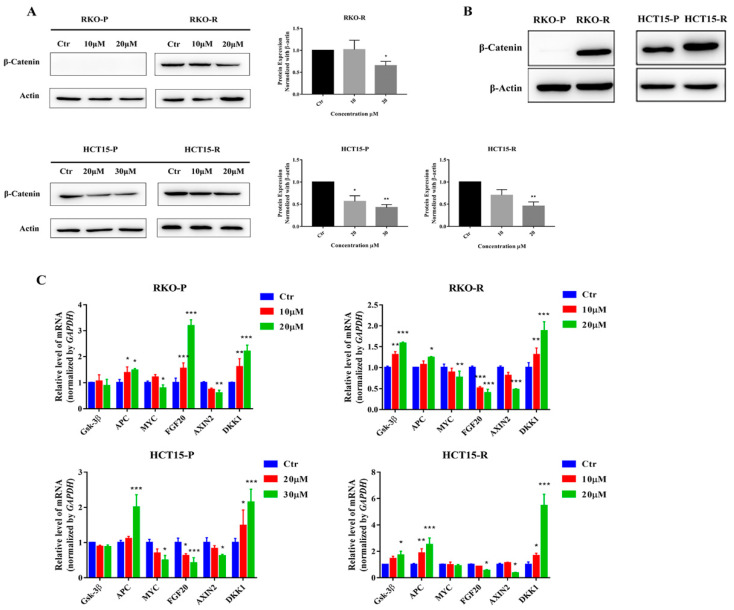
AGE inhibited Wnt signaling pathway in 5-FU-sensitive/resistant CRC cell lines. (**A**): The β-Catenin expression levels were measured and analyzed with β-Actin, and results are shown in the right diagram. As there is almost no expression of β-Catenin protein in RKO-P cells, there are no results for the RKO-P cells. (**B**): The expression levels of β-Catenin protein in the RKO-P/R and HCT15-P/R cells. (**C**): AGE regulated the expression levels of related to Wnt signaling pathway genes, such as *Gsk 3β*, *APC*, *MYC*, *FGF20*, *AXIN2*, and *DKK1*. Results are expressed as mean ± SD (*n* = 3). * *p* < 0.05, ** *p* < 0.01, *** *p* < 0.001, compared with Ctr group.

The excessive activation of the canonical Wnt/β-catenin signaling pathway causes progression in cancer development and chemo-resistance in various cancers, which is closely related to cell proliferation, cell cycle, etc., especially for CRC. Additionally, the β-catenin protein is the most important protein in the Wnt signaling pathway. After 24 h treatment of AGE, the level of β-catenin protein was indeed down-regulated (Figure 6A). Remarkably, there was almost no β-catenin protein in the RKO-P cell (Figure 6B). In order to further confirm the effect of AGE on the Wnt pathway, the upstream genes (*Gsk 3β* and *APC*) and downstream targeted genes (*MYC*, *FGF20*, *AXIN2*, and *DKK1*) were evaluated through an RT-PCR assay in all cell lines (Figure 6C). The AGE up-regulated the *Gsk 3β*, *APC*, and *DKK1*, but there was no significant change in the expression of *Gsk 3β* in RKO-P and HCT15-P cells. For *MYC*, *FGF20*, and *AXIN2* genes, AGE noticeably down-regulated them. However, the expression of the *FGF20* gene was up-regulated in RKO-P cells, and the *MYC* gene showed no significant difference in HCT15-R cells. In summary, AGE can effectively inhibit the Wnt signaling pathway. 

## 3. Discussion

CRC has developed to be a cancer with high morbidity and mortality worldwide. Although improved treatment strategies have been applied in clinics, there is an urgent need to find novel and effective agents for CRC treatment due to the occurrence of side effects and the emergence of drug resistance. Numerous studies have confirmed that many natural plants or compounds possess effective anti-CRC bioactivities and may serve as potential chemotherapeutic agents for CRC treatment [18,19,20], such as alkaloids, polysaccharides, polyphenols, terpenoids, and unsaturated fatty acids, with various structures.

In our previous work, a traditional Chinese medicine *Sanguisorba officinalis* L., including terpenoids, phenols, flavonoids, and some other compounds [7], indicated a significant anti-cancer activity against CRC cells and synergistically enhanced the cytotoxicity of 5-FU against CRC cells [8]. Additionally, it also inhibited the 5-FU resistant cells proliferation (Figure S1). After 48 h of treatment, the IC_50_ values of *Sanguisorba officinalis* L. in RKO-P/R and HCT15-P/R were 148.9 μg/mL, 121.7 μg/mL, 163.6 μg/mL, and 105.2 μg/mL, respectively. Qi found *Sanguisorba officinalis* L. had an activating effect on the key target proteins corticosteroid 11-beta-dehydrogenase isozyme 2, the enzyme encoded by HSD11B2, which was markedly decreased in colorectal cancer tissues and was positively correlated with the overall survival time of patients [21]. The results indicated *Sanguisorba officinalis* L. could promote tissue differentiation in colorectal cancer. Additionally, ziyuglycoside II is one of the most representative and well-known triterpenoids of *Sanguisorba officinalis* L. [22]. In Bai’s research [23], ziyuglycoside II induced apoptosis through the accumulation of reactive oxygen species (ROS), triggered complete autophagic flux, and improved the sensitivity of 5-fluorouracil in colorectal cancer cells. Gallic acid, a major compound of phenols in *Sanguisorba officinalis* L., could bind to ferroptosis-related targets and regulate the expression of corresponding proteins; ferroptosis inhibitors reversed the experimental results, improving CRC by regulating ferroptosis [24]. Another compound named 3,3′,4′-trimethylellagic acid (TMEA), a tannin compound isolated from Sanguisorba officinalis L., executed its anti-cancer activity by inducing apoptosis and inhibiting angiogenesis in CRC cells via the apoptotic and VEGF/PI3K/AKT/mTOR pathways [25]. A type of tannin compound named 1,4,6-Tri-O-galloyl-beta-d-glucopyranose significantly inhibited the Wnt/beta-catenin signaling pathway, up-regulated the protein levels of cleaved caspase-3 and cleaved PARP, as well as the ratio of Bax/Bcl-2 proteins in HT29 cell [26]. Ellagic acid (EA), a natural phenolic constituent, has been shown to exhibit anti-cancer effects. Zhao studied EA targeted to the TGFbeta1/Smad3 pathway on HCT116 cells, which might be potentially used to treat CRC [27]. Additionally, they also found that EA modified a large number of cellular functions, including proliferation, apoptosis, cell cycle, and angiogenesis [28]. Taken together, *Sanguisorba officinalis* L. has a good effect against colorectal cancer.

Therefore, a number of compounds from *Sanguisorba officinalis* L. were determined for their anti-proliferative ability towards CRC cells (Figure S2), especially for 5-FU resistant cells. The compounds had inhibitory effects on CRC cells, including ziyuglycoside I, ziyuglycoside II, and catechin, but not significantly. Among them, a triterpenoid compound named AGE showed good effects to anti-CRC (5-FU-sesntive/resistant) cells, inhibiting cell proliferation in both the short-term and long-term. Moreover, AGE showed lower cytotoxicity to NCM 460 cell (human normal intestinal epithelial cell) than CRC cells, indicating that the side effects of AGE on human normal intestinal epithelial cells are less at the concentration of cancer cell inhibition, which suggested it to be a potential effective agent to treat CRC.

To further clarify the function mechanism of AGE, firstly, a flow cytometry analysis, mitochondrial membrane potential measurement, and Western blotting analysis were applied. The results showed that AGE up-regulated the pro-apoptotic Bax protein expression and down-regulated the anti-apoptotic Bcl-2 protein expression, which tends to tumor growth suppression and sensitizes CRC cells to death. The Bax/Bcl-2 ratio increased, representing an indicator for cells undergoing apoptosis [29]. In addition, the cells’ MMP changes resulted in the release of cytochrome c from mitochondrion to the cytosol, forming the caspase-activating apoptosome platform, which recruited and activated caspase-9. Activated caspase-9 was self-cleaved and activated other downstream effector caspases, such as caspase-3. Subsequently, all activated effector caspases then cleaved a series of protein substrates to initiate apoptosis, leading to the subsequent dismantling of cellular components. Additionally, AGE up-regulated cleaved PARP protein, which also resulted in cells lysis. Taken together, AGE could induce cell death via an intracellular mitochondrial-caspase-dependent apoptosis pathway.

In addition, AGE also initiated cell autophagy in CRC cells. The LC 3Ⅱ/LC 3Ⅰ ratio was increased, which was determined by Western blotting assay. The results showed that autophagy might parallel with apoptosis in executing CRC cell death, which had also been founded in other studies [30,31]. However, in other cases, autophagy can antagonize apoptosis [32]. Inhibitory agents of autophagy or the silencing of autophagy related genes has been shown to potentiate apoptosis signaling in CRC cells or colon cancer-bearing mice treated with DNA disruption drugs, targeted therapy, or other compounds or extracts. In short, the relationship between cell apoptosis and autophagy processes is somewhat complex [10,11], but the interaction between these two pathways plays a pivotal role in CRC cells undergoing pathophysiological changes.

Furthermore, AGE also influenced the cell cycle, reducing the expression of *CCNA2* and *CCND1* genes, which are related to transition through G1/S and G2/M. Thus, the cell cycle of CRC cells was arrested in the G0-G1 phase, which decreased cell proliferation, increased cell apoptosis, and finally induced cell death. These results were consistent with many previous reports [33,34]. Then, which pathway is involved in and mediating the arrest of cell cycle induced by AGE?

The Wnt signaling pathway is known to be closely related to cell proliferation and cell cycle in cancer cells, especially for CRC cells. Additionally, our previous study reported that *Sanguisorba officinalis* L. blocked the Wnt/β-catenin signaling pathway [26]. Therefore, the Wnt signaling pathway may mediate the cell cycle arrest induced by AGE. Thus, the effect of AGE on the Wnt signaling pathways was further verified in this study. In brief, the data showed that AGE up-regulated the *APC* and *Gsk* *3β* gene expression and decreased β-catenin protein expression, which inhibited the abnormal activation of the Wnt pathway. APC is a tumor suppressor with a functional role in the canonical Wnt/β-catenin signaling pathway. Moreover, the APC could bind to β-catenin, thereby reducing the interaction with T cell-specific factor/lymphoid enhancer-binding factor (TCF/LEF) [35]. Additionally, GSK-3β is a key kinase of the Wnt signaling pathway that promotes the degradation of β-catenin protein. Furthermore, the downstream genes related to the Wnt signaling pathway were also down-regulated by AGE, including *MYC*(c-Myc) and *AXIN2*. Additionally, the expression of *DKK1* gene was increased by AGE. This result was similar to Hua’s study, in which increasing *APC* and *DKK1* expression and deceasing β-catenin and *MYC* expression were found [36]. These genes are commonly up-regulated in the abnormally activated Wnt pathway, inducing the occurrence and development of CRC. *MYC* is a proto-oncogene frequently observed in numerous human cancers. Additionally, over-expression of *AXIN2* gene elicited Wnt pathway activation, accelerated cancerization of CRC, and promoted CRC metastasis [37]. In addition, *DKK1* was reported to be an antagonist of the Wnt signaling pathway in CRC [38]. Taken together, AGE down-regulated the transcription of elevated expression genes of *MYC*, *FGF20*, and *AXIN2*, and upregulated the *DKK1* gene expression, suggesting that AGE blocked the Wnt/β-catenin signaling pathway and influenced the gene expression of downstream pathway, which is related to the cell cycle arrest and the cell proliferation inhibition.

The results also showed that the β-catenin protein expression is almost non-existent in the 5-FU-senstitive RKO cell (RKO-P), but higher in the 5-FU-resistant RKO cell (RKO-R), and slightly higher in 5-FU-resistant HCT15 cell (HCT15-R) than that in the 5-FU-senstitive HCT15 cell (HCT15-P). These results are consistent with Kukcinaviciute’s study [39], which indicated that the Wnt/β-catenin signaling pathway in 5-FU-resistant cells is more abnormally activated than 5-FU-sensitive cells. Consequently, the reason that AGE has a stronger effect on drug-resistant CRC cells than sensitive CRC cells may be that the expression of β-catenin protein in the Wnt signaling pathway of drug-resistant cells is more than that of sensitive cells, and AGE could target the Wnt signaling pathway.

## 4. Materials and Methods

### 4.1. Cell Lines and CULTURE

The 5-FU-sensitive/resistant colorectal cancer cells (RKO-R and HCT15-R are the 5-FU resistant cells while RKO-P and HCT15-P are the 5-FU sensitive parental cell lines) were kindly provided by Professor Huanliang Liu from the Sixth Affiliated Hospital, Sun Yat-sen University (Guangzhou, Guangdong, China). The human normal colon epithelial cell (NCM460) was purchased from ATCC. All cells were cultured in DMEM medium (Gibco, Beijing, China), supplied with 10% fetal bovine serum and 1% penicillin-streptomycin (Gibco, Grand Island, NY, USA), in an atmosphere of 5% CO_2_ at 37 °C. 5-FU solution was added into the culture medium for resistant cells to maintain the drug resistance. The cells were maintained in 5-FU-free medium at least 1 week before the experiments.

### 4.2. Chemicals and Reagents

AGE (purity was higher than 98% detected by HPLC) was provided by Professor Chunjuan Yang from College of Pharmacy, Harbin Medical University (Harbin, Heilongjiang, China) and dissolved in DMSO (MP, Solon, OH, USA) to a 20 mM stock solution and stored at −20 °C. 5-FU (Sigma Aldrich, St. Louis, MO, USA) was dissolved in DMSO and stored at −80 °C for long term use. The primary antibodies against Cytochrome C were from Biovision (Milpitas, CA, USA). The anti-Bcl-2, Bax, Caspase-3, PARP, β-Catenin, and β-Actin were purchased from Cell Signaling Technology (Beverly, CA, USA). The anti-LC 3B and Caspase-9 were from ZEN BIO (Chengdu, China). The secondary antibodies were obtained from Millipore Corporation (Temecula, CA, USA).

### 4.3. Cell Viability Assay

Cells were seeded in 96-well plate at a density of 5 × 10^3^ cells per well for 24 h. Then, cells were treated with various doses of AGE. After 24 h, 48 h, and 72 h, CCK-8 (Dojindo, Kyushu, Kumamoto-Ken, Japan) assays were performed to evaluate the cell viability by microplate reader (Omega, Ortenberg, Germany).

### 4.4. Cell Colony Formation Assay

Cells were plated in a 6-well plate (1000 cells/well) and treated with different concentrations of AGE and cultured for 9 days. The medium was changed every 3 days. Then, cells were washed by PBS for twice, fixed in 4% paraformaldehyde (Boster, Wuhan, China) for 20 min and stained with crystal violet (Beyotime, Jiangsu, China).

### 4.5. Hoechst 33258 Staining Assay

Cells were seeded in 12-well plates (2 × 10^5^ cells/well). After attachment, the cells were treated with diverse doses of AGE. After 48 h exposure to drugs, the cells were washed twice with PBS and fixed in 4% paraformaldehyde for 20 min before staining with Hoechst 33258 solution (Beyotime, Jiangsu, China). Cells were photographed by an inverted fluorescence microscope (Olympus, Tokyo, Japan).

### 4.6. Flow Cytometry Analysis of Apoptosis and Cell Cycle

Apoptosis was determined by flow cytometry using an Annexin V-FITC/PI kit (BD Biosciences, Franklin Lakes, NJ, USA). Cells were collected, washed twice with cold PBS after being AGE treated, and stained with a working solution (500 μL binding buffer with 5 μL Annexin V-FITC and 5 μL PI) for 15 min at room temperature in the dark. In the cell cycle assay, after drug treatment, the cells were dispersed and washed with cold PBS before adding pre-cooled 70% ethanol. Then, cells were washed with PBS and incubated with 500 μL PI/RNase solution (Beyotime, Jiangsu, China) for 30 min in the dark. The prepared cellular samples were immediately analyzed on flow cytometry (Temecula, CA, USA).

### 4.7. Mitochondrial Membrane Potential (MMP) Measurement

MMP was detected using a JC-1 mitochondrial membrane potential assay kit (Beyotime, Jiangsu, China). Briefly, cells were washed twice in PBS and incubated with JC-1 staining solution at 37 °C for 20 min after AGE treatment, and then washed twice and resuspended in JC-1 buffer, followed by analysis of JC-1 kit using flow cytometry. The MMP level was measured by mean light intensity of red-green fluorescence.

### 4.8. Western Blot Analysis

Total cell lysates were prepared in a RIPA lysis buffer containing protease inhibitors (Beyotime, Jiangsu, China). The concentrations of total proteins were determined using the Pierce BCA protein assay kit (Thermo Fisher Scientific, Rockford, IL, USA). Then, 30 μg of each cellular sample was loaded in an 8–15% SDS-PAGE gel. The gels were transferred to PVDF films (Millipore, Darmstadt, Germany), blocked with 5% skim milk for 1 h and then blotted with the indicated antibodies at 4 °C overnight. The films were washed three times with TBST, and cultured with HRP-conjugated secondary antibody for 1 h at room temperature before washing a further three times. Then, Super Signal™ West Pico Chemiluminescent Substrate (Thermo Fisher Scientific, Rockford, IL, USA) was used to visualize the protein bands, which were imaged by Automatic Chemiluminescence Image Analysis System (Tanon 5200, Shanghai, China).

### 4.9. Reverse Transcription Quantitative PCR Assay

After being incubated with different AGE solutions, cells were lysed by Trizol reagent (Invitrogen, Carlsbad, CA, USA) to extract the total RNA. The RNA concentration of each sample was determined with the Nano Drop at 260/280 nm (Thermo Fisher Scientific, Rockford, IL, USA), then the total RNA was used to perform the reverse transcription to obtain cDNA according to the manufacture’s protocol of Prime Script RT reagent Kit (Takara, Kusatsu, Shiga, Japan). The cDNA was mixed with reaction reagent of SYBR Premix Ex Tap^TM^ kit (Takara, Kusaatsu, Shiga, Japan) and PCR Forward/Reverse Primer to perform the RT-PCR assay on PCR system (Roche, Indianapolis, IN, USA). The obtained data was analyzed using 2^ (^−^^ΔΔCt^) approach. The genes primers sequences are shown in Table S1.

### 4.10. Statistical Analysis

All experiments in this study were performed at least 3 times, and the obtained data was expressed as mean ± SD. The statistical analysis was performed by one-way ANOVA analysis or *t*-test by GraphPad Prism 7.0 software. *p* < 0.05 was considered as a significant difference.

## 5. Conclusions

In conclusion, the AGE, a triterpene from *Sanguisorba officinalis* L., was first found to have anti-proliferative activity against 5-FU-sensitive/resistant CRC cells, and displayed minimal cytotoxicity to normal cell. In addition, AGE induced cell death through intracellular mitochondrial-caspase-dependent apoptosis pathway and cell autophagy, and inhibited cells proliferation by arresting the cell cycle in the G0-G1 phase through the Wnt signaling pathway, which enhanced the effect of AGE on 5-FU-sensitive/resistant CRC cells. These findings suggest that AGE is a potential anti-cancer agent, for both 5-Fu non-resistant or resistant patients, which deserves further study.

## Figures and Tables

**Table 1:** The IC50 of AGE on CRC cells and normal cells.

Cell	IC_50_ Value (μM)		
	24 h	48 h	72 h
RKO-P	28.96 ± 0.64	18.74 ± 1.92	9.15 ± 0.99
RKO-R	25.12 ± 0.46	14.62 ± 1.89	6.49 ± 1.23
HCT15-P	38.96 ± 1.23	29.82 ± 2.83	17.10 ± 2.29
HCT15-R	30.26 ± 1.59	18.93 ± 1.35	10.99 ± 1.42
NCM460	NA	34.15 ± 2.65	28.18 ± 2.53

## Data Availability

All data generated or analyzed during this study are included in this published article.

## Supplementary Materials

The following are available online, Figure S1: CCK8 assay for the *Sanguisorba officinalis* L extract after cells being treated 48 h., Figure S2: CCK8 assay for the several compounds isolated from *Sanguisorba officinalis* L., Figure S3: 5-FU-sensitive/resistant CRC cell lines (A): CRC cell lines morphology under a microscope (200×); (B): CCK8 assay for the 5-FU after cells being treated 48 h, Table S1: The genes primers sequences of RT-PCR.

## Sample Availability

Samples of the compounds tested in this study are available from the corresponding author Chunjuan Yang.
