# Supplementary material for: A Bioactive Compound from Sanguisorba officinalis L. Inhibits Cell Proliferation and Induces Cell Death in 5-Fluorouracil-Sensitive/Resistant Colorectal Cancer Cells
Source: Molecules. 2021 Jun 24;26(13):3843. doi: 10.3390/molecules26133843 (PMC8270258; doi:10.3390/molecules26133843)
Supplement: Supplementary file 1 [file molecules-26-03843-s001.zip › molecules-1251209-supplementary.pdf]

Supplement

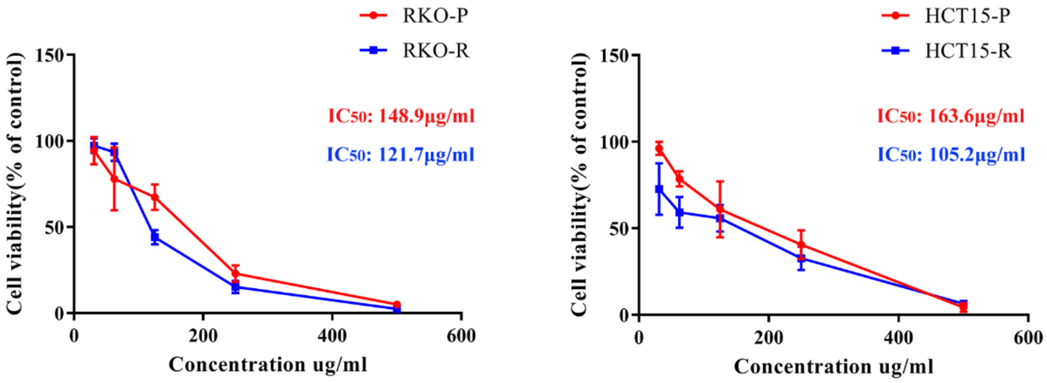

Figure S1. CCK8 assay for the *Sanguisorba officinalis L* extract after cells being treated 48h.

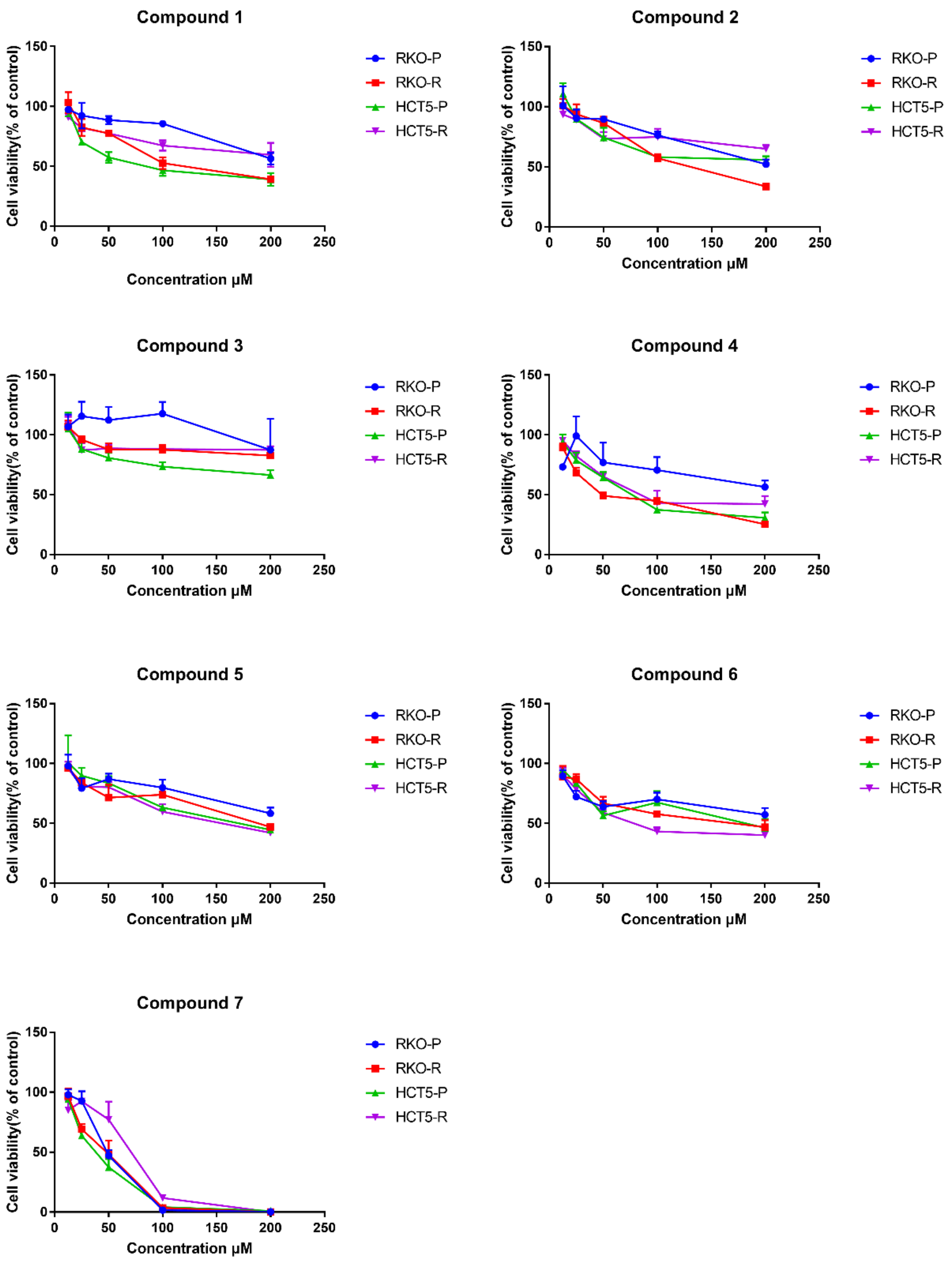

Figure S2. CCK8 assay for the several compounds isolated from *Sanguisorba officinalis L.*

Compound 1: Ziyuglycoside I. Compound 2: Ziyuglycoside II.

Compound 3: Catechin. Compound 4: 3, 4-dihydroxy-5-methoxybenzyl ester.

Compound 5: 1, 4, 6-tri-O-galloyl-β-D-pyran glucose.

Compound 6: Potentillanoside B.

Compound 7: 3β, 19α-dihydroxyurs-12-en-28-oic-acid 28-β-D-glucopyranosyl ester.

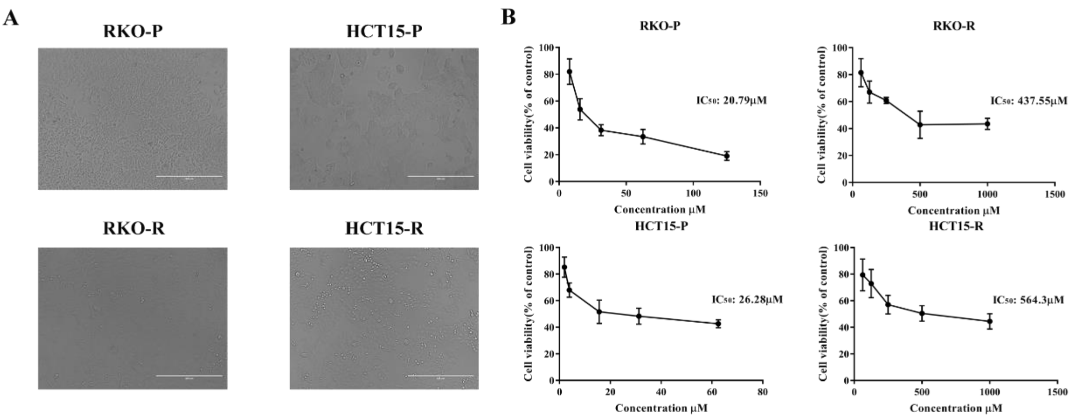

Figure S3. 5-FU-sensitive/resistant CRC cell lines (A): CRC cell lines morphology under a microscope (200×) ; (B): CCK8 assay for the 5-FU after cells being treated 48h.

Table S1. The genes primers sequences of RT-PCR.

	Forward	Reverse
CCNA2<br>*(Cyclin A2)*	5’-AGAAACAGCCA<br>GACATCACTAA-3’	5’-TTCAAACTTTG<br>AGGCTAACAGC-3’
CCND1<br>*(Cyclin D1)*	5’-GTCCTACTTCAA<br>ATGTGTGCAG-3’	5’-GGGATGGTCTCC<br>TTCATCTTAG-3’
BECN1<br>*(Beclin1)*	5’-ATCTAAGGAGCT<br>GCCGTTATAC-3’	5’-CTCCTCAGAGTT<br>AAACTGGGTT-3’
SQSTM1<br>*(P62)*	5’-CCGTCTACAGGTG<br>AACTCCAGTCC-3’	5’-CTCCTCAGAGTT<br>AAACTGGGTT-3’
Gsk 3β	5’- AGGAGAACCCA<br>ATGTTTCGTAT-3’	5’- ATCCCCTGGAAA<br>TATTGGTTGT-3’
APC	5’-TGGAGAACTCAA<br>ATCTTCGACA-3’	5’-ATCTGTCCAGAA<br>GAAGCCATAG-3’
MYC<br>*(c-Myc)*	5’-CGACGAGACCTT<br>CATCAAAAAC-3’	5’-CTTCTCTGAG<br>ACGAGCTTGG-3’
AXIN2	5’-AGGCTAGCT<br>GAGGTGT-3’	5’-AGGCTTGG<br>ATTGGAGAA-3’
FGF20	5’-ATTCATCAGTG<br>TGGCAGTGG-3’	5’-GCTCCCTAAA<br>GATGCATTCG-3’
DKK1	5’-CTGCAAAAATG<br>GAATATGTGT-3’	5’-CTTCTTGTCCT<br>TTGGTGTGA-3’
GAPDH	5’-AGGTCGGAGT<br>CAACGGATTTG-3’	5’-TGTAAACCATGT<br>AGTTGAGGTCA-3’
